# Understanding the socioeconomic costs of dystrophic epidermolysis bullosa in Europe: a costing and health-related quality of life study

**DOI:** 10.1186/s13023-022-02419-1

**Published:** 2022-09-06

**Authors:** A. Angelis, J. E. Mellerio, P. Kanavos

**Affiliations:** 1 Department of Health Services Research and Policy, London School of Hygiene and Tropical Medicine, London, UK; 2 Department of Health Policy and LSE Health, Medical Technology Research Group, London School of Economics and Political Science, London, UK; 3 St John’s Institute of Dermatology, King’s College London and Guy’s and St Thomas’ NHS Foundation Trust, London, UK

**Keywords:** Dystrophic epidermolysis bullosa (DEB), Cost-of-illness (COI), Health-related quality of life (HRQoL), Socioeconomic burden, Europe (EU), Rare diseases

## Abstract

**Background:**

Dystrophic epidermolysis bullosa (EB) is a family of rare genetic dermatological conditions. Recent evidence indicated that in addition to its detrimental implications on patient health-related quality of life (HRQoL), there are substantial socioeconomic cost implications*,* especially regarding direct non-medical costs. This study aims to understand the burden of dystrophic EB (DEB) in Europe, using a primary EB patient-level dataset.

**Methods:**

A bottom-up, cross-sectional, study design was adopted for non-institutionalised patients diagnosed with EB who received outpatient care across EU5 countries: France, Germany, Italy, Spain, and the United Kingdom. A prevalence-based approach was used to estimate resource utilisation from a societal perspective, including direct (medical and non-medical) and indirect costs for patients and caregivers. Patient and caregiver outcomes were obtained using the EQ-5D questionnaire.

**Results:**

A sample of 91 DEB patients was analysed. Overall, average EU5 annual cost per patient was estimated at €53,359, ranging from €18,783 (France) to €79,405 (Germany). Average EU5 annual direct medical costs were estimated at €8357 (15.7% of total), ranging from €5658 (France) to €12,576 (Germany); average direct non-medical costs were estimated at €41,353 (77.5% of total), ranging from €11,961 (France) to €57,000 (Germany); and average indirect costs were estimated at €3649 (6.8% of total), ranging from €1025 (Italy) to €9930 (United Kingdom). Costs varied across patients with different disability but also between children and adults. The mean EQ-5D index score for adult DEB patients ranged between 0.304 (United Kingdom) and 0.541 (Germany), with an EU5 average of 0.456, whereas the mean EQ-5D visual analogue scale score ranged between 47.5 (Germany) and 70.0 (France), with an EU5 average of 61.9. Limitations included potential patient selection bias, recall bias, and exclusion of bandaging and related costs.

**Conclusions:**

The study revealed a substantial socioeconomic burden for DEB in Europe, attributable mostly to high direct non-medical costs, with the majority of patients requiring support from caregivers at home. Compared to the average economic burden of the overall EB patient population, costs for DEB patients are higher across all components of direct medical, direct non-medical and indirect costs.

**Supplementary Information:**

The online version contains supplementary material available at 10.1186/s13023-022-02419-1.

## Background

Epidermolysis bullosa (EB) is a family of rare genetic dermatological conditions. It consists of a group of inherited connective tissue disorders characterised by the absence of a particular cohesion protein in the skin that leads to a defective connection of its outer and inner layers (epidermis and dermis), making the skin fragile [1, 2]. As a result, skin's top layer does not ‘stick’ securely to the layer beneath it and, where the two layers separate, a blister develops. EB can be classified into 4 main types based on the layer of the skin affected: EB simplex (EBS), junctional EB (JEB), dystrophic EB (DEB), and Kindler EB (KEB) [1, 3, 4]. Each type can be further subdivided at molecular level, according to the structural gene targeted by the mutation, and clinically as generalised (with widespread sites of blistering) or localised (where blistering is localised to the hands, feet or lower legs) [1]. The severity of the disease can vary from benign to life-threatening. Symptoms include skin fragility, skin blistering after mild friction or trauma, and internal blistering of the mucous membranes and/or internal organs, a severe symptom that often leads to a shorter lifespan [3, 5–7]. The prevalence of DEB is estimated to range between 3.3 and 5.7 per million people [8, 9], accounting for about 30% of the total EB population [10, 11].

Currently there is no cure for EB with clinical management focusing on treating the symptoms of the disease [12] and providing psychological support and follow-up [13]. A variety of topical preparations and dressings are used to protect skin and cover wounds [14], and ongoing surveillance is often necessary to monitor for the occurrence of skin squamous cell carcinomas[15]. Mucosal and internal complications such as oesophageal strictures, dental disease, corneal erosions, constipation, renal impairment and cardiomyopathy are also prevalent. Multidisciplinary management of these complications forms standard of care for EB in many countries, including the EU5 among other European countries [16, 17].

The impact of the condition on patient health-related quality of life (HRQoL) has been explored both from a qualitative and a quantitative perspective [2, 3, 5–7, 18–22]. Qualitative results have revealed a high prevalence of psychosocial problems and psychiatric symptoms [3, 20]. This highlights the importance of a multidisciplinary approach that provides the appropriate psychological and peer support [2] in tandem with pain management and nursing support [21]. More quantitative approaches have assessed different HRQoL dimensions of EB patients using a range of instruments including the Dermatology Life Quality Index (DLQI) and the Children's Dermatology Life Quality Index (CDLQI) [5], the QOLEB questionnaire [6, 22], the Short Form-36 (SF-36), Skindex-29, General Health Questionnaire-12 (GHQ-12) and EuroQOL 5 dimensions (EQ-5D) questionnaires [7]. Others have also looked at the burden to carers [7] and parents [19]. Overall, they have shown impaired HRQoL for both the patients [5, 7, 18] and their carers [7, 19]. An earlier European study indicated that, in addition to its negative implications on patient HRQoL, there are substantial socioeconomic costs, including direct non-medical costs [23].

The aim of the study is to better understand the burden of dystrophic EB (DEB) patients in Europe. The study focuses on the analysis of primary patient-level data from DEB patients, leveraging an earlier European project (BURQOL-RD) with a total sample of 184 EB patients across the EU5 (France, Germany, Italy, Spain, and the United Kingdom) [23].

## Methods

### Study design and patient sample

The study’s patient enrolment and data collection methodology are publicly available [23]. As part of the BURQOL-RD (Social economic burden and health-related quality of life in patients with rare diseases in Europe) initiative [24, 25], patient-level primary data was collected across European Member States. The aim was to estimate the social and economic cost burden of EB in terms of direct medical, direct non-medical and loss of productivity (i.e., indirect costs), and also report HRQoL impairment for both patients and their caregivers. The countries involved in the DEB patient data collection were France, Germany, Italy, Spain and the United Kingdom (UK).

A bottom-up, cross-sectional design was adopted that targeted non-institutionalised patients diagnosed with EB who received outpatient care. Because of the lack of patient registries at national level, patients were recruited with the assistance of national EB associations across the study countries based on their membership status. The survey was completely anonymous, and patients were contacted by their patient association. Patient eligibility criteria included: (i) EB diagnosis, (ii) non-institutionalised status and (iii) membership with the respective national EB patient association. All questionnaire responses received contained no identification information (i.e., name, address/postcode, e-mail, telephone). All patients and caregivers were informed about the study’s objective and data confidentiality arrangements and were then asked to indicate their understanding of the study conditions and their agreement to participate. The original study protocol received an exemption from the London School of Economics (LSE) Research Ethics Committee.

Following the identification of patients who fulfilled the eligibility criteria, patient associations administered and distributed the questionnaires electronically or by post between September 2011 and April 2013. The recruitment period did not exceed six months in any of the study countries. Demographic, clinical and resource use data were collected from EB patients and their caregivers. The generic questionnaire instrument used for data collections is listed in Additional file 1.

### Costing methodology

The disease prevalence approach was adopted from a societal perspective to estimate the amount of resources used and costs incurred. In a sample of patients within a year, this approach considered: all direct medical resources used for prevention, treatment, and rehabilitation; other non-medical resources used (formal and informal care); and any labour productivity lost as a consequence of the illness. Prevalence-based cost-of-illness analysis has the advantage of incorporating measurements of total annual medical expenditure, which is particularly relevant for chronic conditions requiring long-term treatment such as EB. In this context, a bottom-up costing approach was used to estimate total and average annual costs [26]. Original costing data estimated for the year 2012 were inflated to year 2020 using IMF Country Harmonized Indexes. More precisely, direct medical costs were inflated using the Health Index whereas direct non-medical costs and indirect costs were inflated using general Consumer Price Index [27]. In both cases, the average annual estimate for each country was calculated for years 2012 and 2020, using the formula below:

$$Overall\;cost = Direct\;costs + Indirect\;costs,$$

where *Direct costs* = direct medical costs (i.e. drug costs, diagnostic test costs, physician visit costs, hospitalisation costs, material costs, medical transportation costs) + direct non-medical costs (i.e. social health services costs, professional carer costs, transportation costs, main informal care costs, other informal carer costs); *Indirect costs* = patient productivity loss + patient early retirement costs.

Data on resource utilisation were collected for each patient and, where appropriate, for the caregiver. To estimate resource utilisation, the questionnaire collected information for 6-months prior to the study, which was considered as an appropriate recall period (12 months for hospital admissions), and then data was extrapolated to the full year. Productivity losses were calculated using patient and caregiver data collected on working time reductions (temporary sick, permanent sick leave, early retirement). Non-professional caregivers were also asked about informal care time.

### Direct medical costs

Direct medical costs were derived from medical utilisation. The cost of resources used by patients was calculated based on the relevant unit costs and the average utilisation per patient in the sample. Information about the number of hospital admissions, emergency visits and volume of outpatient care (rehabilitation, medical tests and examinations, visits to health professionals and home medical care) was collected from the questionnaires.

Unit costs were obtained from different European databases of medical costs and any remaining data gaps were filled in using additional publicly available resources (see Additional file 2). To derive the annual cost per patient, unit costs were multiplied by the respective resource quantities, using 2012 as the reference year, and then inflated to 2020 figures using IMF inflation indexes as described above [27]. Similarly, resource utilisation information relating to the use of prescription drugs and medical support devices was obtained from the questionnaires. For prescription drugs, when no information concerning the number of units per pack was available, the largest dispensing pack was assumed. Prescription drug unit costs were also obtained from government databases (see Additional file 2), whereas unit costs for medical support devices were obtained from major electronic commerce websites.

### Direct non-medical costs

Direct non-medical costs were quantified by aggregating three components: non-medical transportation, social care services (formal care), and caregiver time (informal care provided by non-professional caregivers, e.g., relatives, friends, neighbours, etc.). Informal care concerned the amount of time spent helping the patient with their basic activities of daily living (ADL), and the time spent helping with necessary instrumental activities of daily living (IADL), using a recall method. As a conservative criterion, and for preventing conjoint production, the time of care was censored to a maximum of 16 hours (h) per day (i.e., total of 114 h per week) when the time of care reported exceeded this limit. To derive a cost for the amount of care provided by informal caregivers, the proxy good method was used: informal caregiver time was valued as if their services were provided by a professional [28, 29]. Data on formal (i.e., paid) care provided by professional caregivers and other social services were obtained from the questionnaires and reported in the relevant category.

### Productivity losses

Productivity losses were accounted for by converting physical units (i.e., days of sick leave and early retirement) into monetary units using the human capital approach [30]. Worker gross average earnings were used to proxy productivity losses (see Additional file 2). Therefore, calculations were based on average gross wage figures in the Wage Structure Surveys as provided by the National Statistics Institutes of the study countries. Annual labour productivity losses were estimated for the year 2012, which were then inflated to 2020 as described above.

### Patient and caregiver HRQoL outcomes

Patient and caregiver outcomes were obtained via the EuroQoL EQ-5D-3L [31], the Barthel index (BI) [32] and the Zarit burden interview scale [33]. The EQ-5D-3L is a generic HRQoL instrument commonly used in economic evaluations and routinely included in health technology assessment (HTA) evaluations. The EQ-5D-3L consists of two parts and has five dimensions (mobility, self-care, everyday activities, pain/discomfort, and anxiety/depression). In the first part, utilities are assigned a score on a scale from 0 to 1 (elicited using the Time Trade-Off tariff), where 0 corresponds to death and 1 corresponds to perfect health; however, negative values are also possible, i.e., worse than death states. The second part of the EQ-5D-3L consists of a vertical 20-cm, 0–100 visual analogue scale (VAS), where 0 represents the worst and 100 represents the best imaginable health state. Respondents mark a point on the scale to reflect their overall perception of health on the day of the interview. Evaluations of these health states have been reported for the general population across countries.

The BI is widely used to assess physical disability. It measures a person’s ability to perform ten basic ADLs and produces a quantitative estimate of the subject’s degree of dependence. Total possible scores range either from 0 to 20 or from 0 to 100, with lower scores indicating increased disability. All scores were converted into the 0–100 range. A score of 91–99 shows mild dependence, 61–90 moderate dependence, 21–60 severe dependence and < 21 complete dependence. For the analysis, patients were grouped into two categories: lower (i.e., no or mild) disability, defined as having a BI score between 91 and 100, and higher (i.e., moderate, severe, or complete) disability, for BI scores lower than 91.

Lastly, the Zarit Burden Interview scale (22-item version) measures the subjective burden among caregivers. Each item is a statement that the caregiver is asked to respond to using a five-point scale, with options ranging from 0 (never) to 4 (nearly always). The total score ranges from 0 to 88, where scores under 21 correspond to little or no burden, and scores over 61 represent severe burden.

## Results

Out of the total sample of 184 EB patients (37 France, 15 Germany, 35 Italy, 54 Spain, and 43 UK), 91 patients were classified with a dystrophic diagnosis corresponding to the analysis sample (14 France, 4 Germany, 26 Italy, 32 Spain, and 15 UK). The baseline characteristics of the patients are summarised in Table 1. Out of the study sample of 91 (100%) DEB patients, 70 (76.9%) had a generalised DEB diagnosis and 21 (23.1%) had a localised DEB diagnosis. More than half of participants were adults (n = 50) and their average age was 36.3 years, with the lowest average observed in Italy (26 years, n = 15) and oldest average observed in Germany (49 years, n = 2). Mean age for children in the sample (n = 41) was 6.8 years, with the lowest average observed in UK (4.8 years, n = 4) and oldest average observed in Italy (8.8 years, n = 11). There was a higher prevalence of female patients in the sample, accounting for 61.5% of respondents on average, with Germany having females only (n = 4). In contrast to the total EB sample in which only the minority (about 40%) required a caregiver, most patients with a DEB diagnosis (60.4%, n = 55 of 91) required a caregiver.

**Table 1:** Demographic characteristics of study participants (patients = 91, carers = 55)

	France	Germany	Italy	Spain	UK	Total/average
**Patients**						
No.	14	4	26	32	15	91
Sex, females %	57.1	100.0	57.7	59.4	66.7	61.5
All patients, average age						
Years	24.6	28.8	18.7	21.1	31.5	23.0
SD	23.3	24.0	10.6	18.0	20.5	18.1
Adults, No.	7	2	15	15	11	50
Adults, %	50.0	50.0	57.7	46.9	73.3	54.9
Adults, average age						
Years	42.1	49.0	26.0	38.7	41.3	36.3
Adolescents, No.	7	2	11	17	4	41
Adolescents, %	50.0	50.0	42.3	53.1	26.7	45.1
Adolescents, average age						
Years	7.1	8.5	8.8	5.6	4.8	6.8
Disease subtype, %						
Dystrophic generalised	57.1	100.0	88.5	81.3	60.0	76.9
Dystrophic localised	42.9	0.0	11.5	18.8	40.0	23.1
**Caregivers (main)**						
No.	5	2	19	23	6	55
Sex, males, %	0.0	0.0	21.1	17.4	16.7	16.4
Sex, females %	100.0	100.0	78.9	82.6	83.3	83.6
Average age						
Years	37.5	44.0	38.2	47.2	46.4	43.3
SD	8.1	12.7	19.3	15.3	11.3	16.1
Relationship to patient, %						
Parent	40.0	100.0	26.3	87.0	50.0	58.2
Other relative	20.0	0.0	57.9	8.7	0.0	25.5
Partner or other	20.0	0.0	5.3	4.3	33.3	9.1
Employment status, %						
Employed	40.0	100.0	31.6	43.5	33.3	40.0
Retired	0.0	0.0	15.8	21.7	0.0	14.5
Houseworker	20.0	0.0	26.3	30.4	50.0	29.1
Other	20.0	0.0	15.8	4.3	0.0	9.1
Average dedication						
Hours per week	27.8	108.8	59.6	81.8	73.0	69.0
SD	37.7	4.5	36.2	32.5	52.3	39.4

The average age of caregivers, for patients that had one (60.4%, n = 55), was 43.3 years and the majority (83.6%, n = 46 of 55) were female. More than half of the caregivers across the study sample were parents to the patients (58.2%), followed by other family relatives (25.5%) and partners or others (9.1%) (these do not add up to 100% because of missing values). Across all countries, a substantial proportion of caregivers were in paid employment other than caregiving (40.0%), with a considerable proportion being involved with domestic activities (29.1%) and a smaller proportion being retired (14.5%) or other (9.1%) (these do not add up to 100% because of missing values). The total average time spent by the main caregiver was estimated at 69.0 h per week, ranging between 27.8 (France, n = 5) and 108.8 h per week (Germany, n = 2).

### Direct and indirect costs

Across the total DEB patient sample (n = 91), the overall average annual cost per patient across all countries was estimated at €53,359 (SD €52,714) in 2020 prices. More specifically, overall annual costs per patient was estimated at €18,783, €79,405, €56,483, €66,823, and €44,546, for France, Germany, Italy, Spain, and the UK respectively (Table 2). A subgroup analysis for localised versus generalised patients is also provided separately in the Additional file (see Additional file 3: Table S1 and Table S2).

**Table 2:** Average annual costs per patient (SD), all patients (n = 91, 2020 €)

	Average		France		Germany		Italy		Spain		United Kingdom	
	Mean	SD	Mean	SD	Mean	SD	Mean	SD	Mean	SD	Mean	SD
Drugs	1104	1711	42	103	60	77	3357	1611	356	627	66	76
Tests	216	407	124	109	131	218	74	112	276	421	446	719
Visits	2651	4099	1338	1881	5240	4084	2288	2099	2646	5256	3825	5172
Hospitals	3228	7630	2054	5154	6026	9564	4149	8819	3534	8632	1326	4102
Material	1085	1601	2087	1851	617	1101	614	1309	424	1403	2502	992
HC transport	73	389	13	47	502	581	0	0	122	603	35	135
**Direct medical**	8357	10,194	5658	7848	12,576	14,221	10,482	10,441	7357	11,608	8201	7169
Social health service	1583	5690	3056	9562	7699	15,398	1895	5072	662	1998	0	0
Professional carer	581	3805	233	872	0	0	568	2007	0	0	2323	8996
Non-HC transport	121	270	204	499	466	604	90	177	95	145	57	96
Main informal carer	29,045	34,656	5561	14,030	40,237	46,502	28,823	34,748	41,756	34,614	21,246	35,264
Other informal carer	10,024	18,998	2907	8440	8598	14,984	13,600	21,696	13,802	22,718	2790	7666
**Direct non-medical**	41,353	48,079	11,961	32,177	57,000	69,348	44,976	48,225	56,316	49,583	26,415	39,526
Productivity loss	274	1808	1164	4356	0	0	86	439	170	961	66	255
Early retirement	3374	9713	0	0	9829	19,658	939	2651	2980	8081	9864	16,932
**Indirect**	3649	9784	1164	4356	9829	19,658	1025	2656	3150	8073	9930	16,893
**TOTAL**	53,359	52,714	18,783	39,323	79,405	72,302	56,483	49,766	66,823	54,753	44,546	48,392

**Fig. 1:**
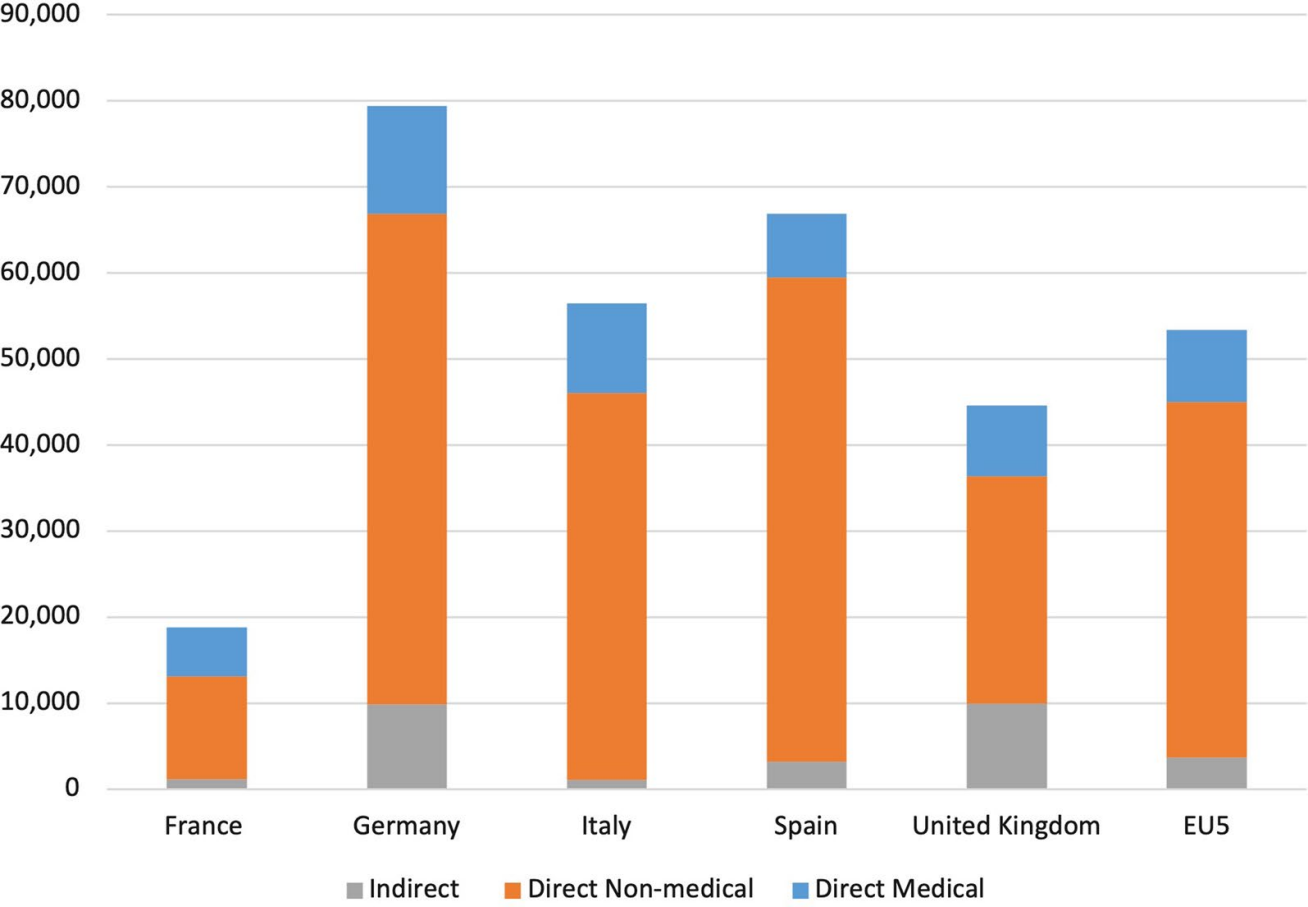
Mean annual costs for all dystrophic epidermolysis bullosa patients across countries (2020, €)

Out of the overall average annual cost per patient across countries (€53,359), 15.7% accounted for direct medical costs (€8357), 77.5% accounted for direct non-medical costs (€41,353), and 6.8% accounted for indirect costs (€3649); the largest cost components were direct non-medical costs across all countries (Fig. 1).

**Fig. 2:**
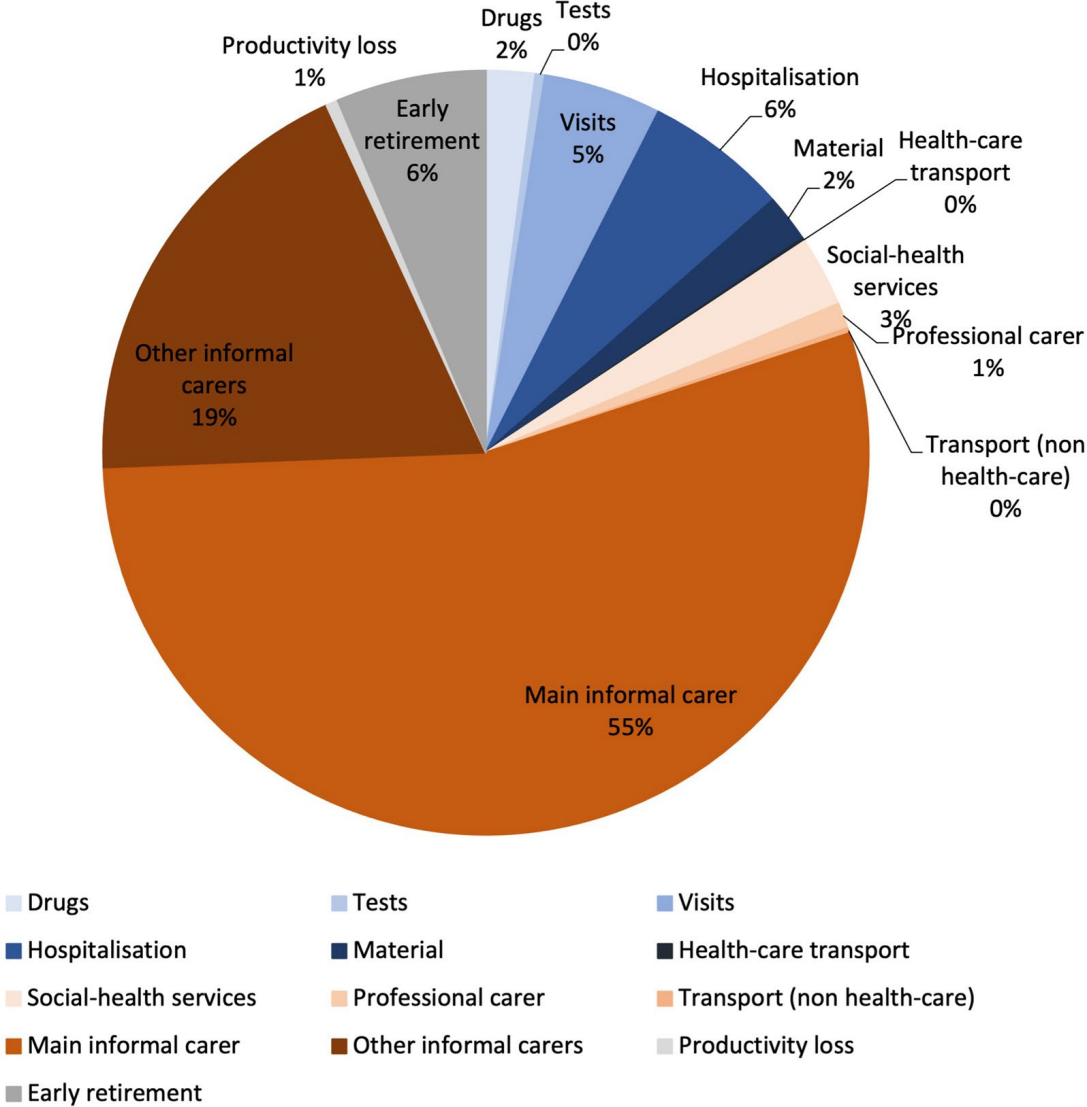
Overall mean annual cost per dystrophic epidermolysis bullosa patient broken down by type of cost (2020, €)

More precisely, out of the average €8357 direct medical costs, drugs corresponded to 2.1% (€1104), tests to 0.4% (€216), outpatient visits to 5.0% (€2651), hospitalisations to 6.0% (€3228), other material to 2.0% (€1085), and medical transportation to 0.1% (€73) of the total cost respectively (Fig. 2). Out of the average €41,353 direct non-medical costs, social health services corresponded to 3% (€1583), professional care to 1.1% (€581), transportation (non-medical) to 0.2% (€121), main informal care to 54.4% (€29,045), and other informal care to 18.8% (€10,024) of the total cost respectively (Fig. 2). The majority of direct non-medical costs was due to informal care (main informal together with other informal), capturing on average almost three quarters of the total cost (73%). With the exception of the UK, in all countries professional care represented a minor proportion of total costs, in contrast to informal care which represented a major proportion of costs across countries (Table 2). Out of the average €3649 indirect costs, patient productivity loss corresponded to 0.5% (€274) and patient early retirement to 6.3% (€3374) of the total costs, respectively (Fig. 2). Indirect costs were a minor item in all countries with the exception of Germany and the UK.

**Fig. 3:**
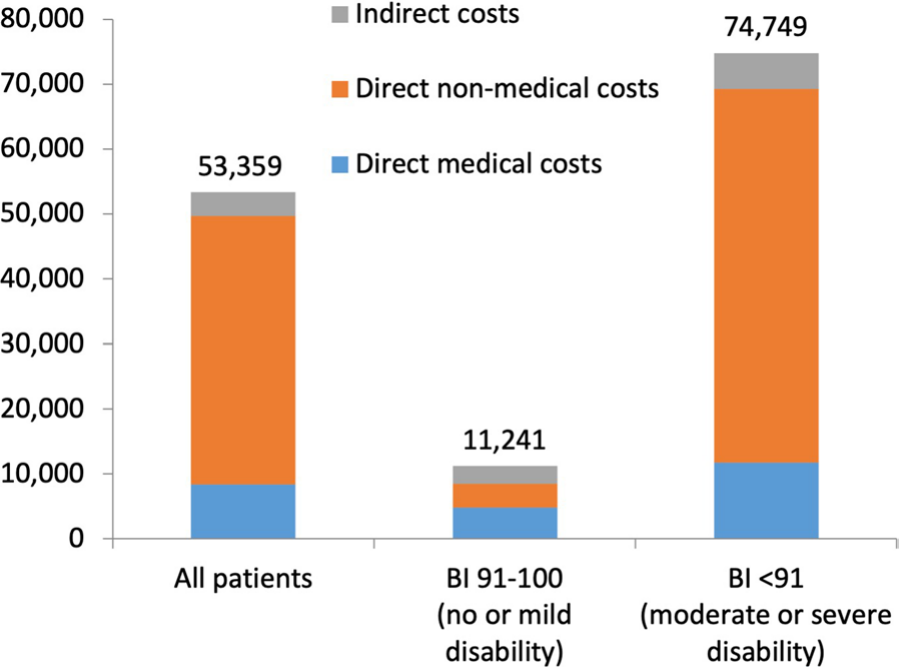
Direct medical, direct non-medical and indirect costs for all patients, patients with lower disability (BI 91–100), and patient with higher disability (BI < 91) (2020, €)

Patients with no or mild disability (BI score 91–100) had an average annual cost of €11,241 (direct medical costs of €4784, direct non-medical costs of €3725, indirect costs of €2732) whereas patients with moderate or severe disability (BI score < 91) had a higher average annual cost of €74,749 (direct medical costs of €11,786, direct non-medical costs of €57,523, indirect costs of €5440) (Fig. 3).

For the adult population (n = 50), overall average annual cost per patient across all countries was estimated at €33,211, ranging from €9961 in France (n = 7) to €44,060 in Spain (n = 15) (Table 3). Average direct medical costs per adult patient across countries was estimated at €7929, ranging from €3335 in France (n = 7) to €13,630 in Italy (n = 15). Average direct non-medical costs per adult patient across countries was estimated at €19,017, ranging from €142 in Germany (n = 2) to €32,196 in Spain (n = 15). Average indirect costs per adult patient across countries was estimated at €6265, ranging from €1777 in Italy (n = 15) to €19,658 in Germany (n = 2) (Table 3). Overall, direct non-medical costs and direct medical costs represented on average 57% and 24% of total costs (€33,211) respectively, with the remaining 19% attributable to indirect costs.

**Table 3:** Average annual costs per patient, adult patients (n = 50, 2020 €)

	Average		France		Germany		Italy		Spain		United Kingdom	
	Mean	SD	Mean	SD	Mean	SD	Mean	SD	Mean	SD	Mean	SD
Drugs	1151	1871	83	138	5	7	3550	1870	427	892	53	78
Tests	254	496	136	112	229	323	101	139	379	550	437	828
Visits	2157	3420	509	551	4965	5884	2874	2374	829	1207	4101	5906
Hospitals	3083	7634	822	2174	2009	2841	6063	11,218	2919	7478	1720	4780
Material	1250	1749	1784	1785	34	3	1043	1611	566	2037	2459	1125
HC transport	34	154	0	0	476	673	0	0	23	87	48	158
**Direct medical**	7929	10,427	3335	3376	7717	9731	13,630	12,750	5143	10,794	8818	8208
Social health service	1335	3876	215	569	0	0	3265	6418	1352	2797	0	0
Professional carer	936	4955	0	0	0	0	985	2598	0	0	3167	10,505
Non-HC transport	96	166	100	171	142	139	111	223	85	122	47	75
Main informal carer	11,450	21,188	2624	5107	0	0	11,031	14,707	20,074	28,670	8293	24,461
Other informal carer	5200	14,314	1359	3129	0	0	6178	13,354	10,686	22,458	0	0
**Direct non-medical**	19,017	32,209	4298	8716	142	139	21,569	23,361	32,196	48,113	11,508	25,541
Productivity loss	471	2359	2328	6160	0	0	149	578	363	1404	90	298
Early retirement	5794	12,206	0	0	19,658	27,801	1627	3369	6358	11,027	13,451	18,663
**Indirect**	6265	12,206	2328	6160	19,658	27,801	1777	3341	6721	10,891	13,541	18,594
**TOTAL**	33,211	42,645	9961	10,176	27,517	37,671	36,976	34,671	44,060	57,418	33,867	46,441

For the paediatric population (n = 41), overall average annual cost per patient across all countries was estimated at €69,559, ranging from €27,605 in France (n = 7) to €131,293 in Germany (n = 2) (Table 4). Average direct medical costs per paediatric patient across countries was estimated at €7584, ranging from €6189 in Italy (n = 11) to €17,435 (n = 2) in Germany. Average direct non-medical costs per paediatric patient across countries was estimated at €61,975, ranging from €19,624 in France (n = 7) to €113,859 in Germany (n = 2) (Table 4). Overall, direct non-medical costs represented on average 89% of total costs (€69,559), with the remaining 11% attributable to direct medical costs.

**Table 4:** Average annual costs per patient, paediatric patients (n = 41, 2020 €)

	Average		France		Germany		Italy		Spain		United Kingdom	
	Mean	SD	Mean	SD	Mean	SD	Mean	SD	Mean	SD	Mean	SD
Drugs	864	1399	0	0	114	75	3094	1208	294	238	105	65
Tests	139	217	111	114	34	13	36	45	184	245	470	353
Visits	2777	4575	2166	2400	5515	3887	1488	1380	4249	6813	3066	2741
Hospitals	2835	7124	3287	7020	10,044	14,204	1540	2294	4076	9736	243	485
Material	864	1316	2391	2005	1200	1509	31	36	298	383	2620	596
HC transport	105	522	25	66	529	748	0	0	210	825	0	0
**Direct medical**	7584	9539	7981	10,462	17,435	20,436	6189	3135	9312	12,266	6503	3170
Social health service	1737	6919	5896	13,377	15,398	21,777	27	88	52	216	0	0
Professional carer	71	481	466	1234	0	0	0	0	0	0	0	0
Non-HC transport	153	347	309	696	790	809	62	84	104	166	84	153
Main informnal carer	45,768	36,421	8498	19,502	80,474	3347	53,084	39,919	60,888	27,794	56,864	38,758
Other informal carer	14,246	21,295	4455	11,788	17,197	19,439	23,720	27,053	16,552	23,271	10,463	12,930
**Direct non-medical**	61,975	50,927	19,624	45,059	113,859	38,676	76,893	55,747	77,597	41,367	67,411	45,396
Productivity loss	0	0	0	0	0	0	0	0	0	0	0	0
Early retirement	0	0	0	0	0	0	0	0	0	0	0	0
**Indirect**	0	0	0	0	0	0	0	0	0	0	0	0
**TOTAL**	69,559	55,103	27,605	55,364	131,293	59,112	83,082	56,212	86,909	44,825	73,914	46,593

### Patient and caregiver HRQoL outcomes

In terms of HRQoL, the mean EQ-5D index score for adult patients was 0.456 (n = 46, SD = 0.328), and ranged from 0.304 (UK, SD = 0.449) to 0.541 (Germany, SD = 0.490) (Table 5). The mean EQ-5D VAS score for adult patients was 61.9 (n = 46, SD = 23.9) and ranged from 47.5 (Germany, SD = 3.5) to 70 (France, SD = 17.3) (Table 5). These point estimate scores are markedly lower than scores reported in the general population across the study countries [34]. In terms of adolescent patients, the mean EQ-5D VAS score was 54.8 (n = 20, SD = 18.2) and ranged from 46.4 (Italy, SD = 11.8) to 62.9 (Spain, SD = 19.8).

**Table 5:** Health-Related Quality of Life characteristics of study participants

	France	Germany	Italy	Spain	United Kingdom	Average
**EQ-5D Index Score**						
Adult patients (n = 46)						
Mean	0.528	0.541	0.469	0.490	0.304	0.456
SD	0.285	0.490	0.262	0.317	0.449	0.328
General population						
Mean	0.91	0.91	0.93	0.91	0.91	0.914
SD	0.16	0.16	0.15	0.16	0.16	0.158
Main caregivers (n = 46)						
Mean	0.720	0.855	0.787	0.726	0.713	0.749
SD	0.243	0.077	0.318	0.296	0.071	0.277
General population						
Mean	0.91	0.91	0.91	0.85	0.91	0.898
SD	0.16	0.16	0.16	0.25	0.16	0.178
**Visual Analog Scale**						
Adult patients (n = 46)						
Mean	70.0	47.5	64.0	61.7	56.2	61.9
SD	17.3	3.5	16.8	28.6	31.2	23.9
General population						
Mean	86.6	86.6	86.8	86.6	86.6	86.6
SD	13.8	13.8	13.8	13.8	13.8	13.8
Main caregivers (n = 46)						
Mean	76.7	67.5	75.4	73.9	75.0	74.3
SD	17.6	10.6	16.1	18.5	14.7	16.6
General population						
Mean	86.6	86.6	86.6	82.0	86.6	85.7
SD	13.8	13.8	13.8	18.2	13.8	14.7
Adolescent patients (n = 20)						
Mean	51.7	60.0	46.4	62.9	57.5	54.8
SD	23.6	0.0	11.8	19.8	31.8	18.2
**Zarit scale (n = 49)**						
Mean	28.0	46.5	32.4	30.7	24.0	31.0
SD	25.7	9.2	13.3	12.3	9.2	13.7
**Barthel Index (n = 65)**						
Mean	94.0	81.7	69.3	77.5	80.0	78.1
SD	14.1	16.1	27.7	19.8	21.7	22.9

Over three quarters of caregivers (n = 46, 83.6%) completed the HRQoL portions of the questionnaire. The mean EQ-5D index score for caregivers was 0.749 (SD = 0.277), and ranged from 0.713 (UK, SD = 0.071) to 0.855 (Germany, SD = 0.077) (Table 5). The mean EQ-5D VAS score for caregivers was 74.3 (SD = 16.6) and ranged from 67.5 (Germany, SD = 10.6) to 76.7 (France, SD = 17.6) (Table 5). When looking at generalised vs localised patients, mean overall adult EQ-5D index scores were 0.456 (SD = 0.330) versus 0.454 (SD = 0.339), whereas mean overall adult VAS scores were 62.39 (SD = 23.2) versus 58.5 (SD = 27.3) (adolescent VAS scores were 54.8 (SD = 18.2) vs. n/a).

The average BI score of patients represented moderate dependence at 78.1 (n = 65, SD = 22.9) (Table 5). The average BI scores of patients from Germany, Italy, Spain and UK reflected moderate dependence (81.7, 69.3, 77.5 and 80.0, respectively), whereas the average BI score of patients from France reflected mild dependence (94.0, SD = 14.1).

The burden for caregivers was moderate across all countries with average Zarit scale score of 31.0 (n = 49, SD = 13.7) and more specifically with average Zarit scale scores of 28.0, 46.5, 32.4, 30.7 and 24.0 for France, Germany, Italy, Spain and UK (Table 5).

## Discussion

This study adds to the existing cost-of-illness literature of rare diseases by focusing on the socioeconomic implications of DEB, further elucidating the economic burden and impact on HRQoL for patients and caregivers.

The disease has a substantial impact on the HRQoL of patients and their caregivers across all study countries. The average EQ-5D index score for adult DEB patients ranged between 0.304 (UK) and 0.541 (Germany), with the overall average across EU5 being 0.456. Similarly, the adult EQ-5D VAS scores ranged between 47.5 (Germany) and 70 (France), with the overall average across countries being 61.9. Overall, patient HRQoL varied across countries and was significantly lower than general population reference values. Furthermore, the HRQoL of caregivers was markedly lower than the general population values, as evident by the EQ-5D index and VAS scores of 0.749 and 74.3, compared to 0.898 and 85.7 respectively. Therefore, our results indicate that DEB patients, and by extension their caregivers, have lower HRQoL than the general population.

Taking into consideration a systematic review of the literature analysing 16 studies reporting EQ-5D measures for patients with plaque psoriasis, psoriatic arthritis and other skin conditions (ranging on average from 0.5 to 0.82), reveals that DEB has a more severe impact on HRQoL [35]. These findings are confirmed by a more recent systematic literature review and meta-analysis on psoriatic patients, demonstrating that DEB has a more detrimental impact on HRQoL [36]. Beyond the disease impact on overall HRQoL, it is important to highlight the effect on lower productivity and employment for both patients and caregivers.

The study highlights the importance of taking into consideration the wider economic consequences of rare diseases such as DEB and interpreting the findings from an international viewpoint. In our analysis, the findings provide insights about the distribution of DEB costs across European countries showing that, using 2020 prices, the estimated average annual costs were €18,783, €79,405, €56,483, €66,823, and €44,546, for France, Germany, Italy, Spain, and the UK, respectively. In terms of the different cost components’ relative contribution, direct non-medical costs comprised the largest share of overall costs at an average of 77.5% per patient across countries, ranging from 59.3% (UK) to 84.3% (Spain). Direct medical costs comprised the second largest share of overall costs at an average of 15.7% per patient across countries, ranging from 11% (Spain) to 30.1% (France). Finally, indirect costs comprised the smallest share of overall costs at an average of 6.8% per patient across countries, ranging from 1.8% (Italy) to 22.3% (UK). These differences across countries are possibly caused due to several factors, including differences in utilisation (of medical and non-medical services), differences in unit costs, differences in clinical guidelines, and differences in patient sample characteristics.

Limited empirical cost-of-illness evidence exists on EB and even less on DEB. In terms of average costs for the EB general population (not specific to any sub-type), the average annual cost per patient across eight European countries was estimated at €31,390, out of which €23,483 (74.8%) corresponded to direct non-medical costs, €5646 (18.0%) corresponded to direct medical costs, and €2261 (7.2%) corresponded to indirect costs (using 2012 prices) [23]. For the EU5 countries, the average annual costs per patient were estimated at €14,931, €46,116, €49,233, €43,137, €19,758 for France, Germany, Italy, Spain, and the UK, respectively. Therefore, although the relative contribution of the three main cost components for DEB patients is very similar to the overall EB population, DEB cost levels are substantially higher.

The only other published study on the economic burden of EB with more than 50 patients corresponds to a recent US study investigating the challenges of patients (n = 63) and caregivers (n = 93) for simplex, junctional, and dystrophic (dominant and recessive) EB [37]. Around a fifth (19%) of patients and a quarter (26%) of caregivers reported a visit to an emergency department in the last 12 months, and although most of the patients (over 95%) had healthcare coverage, most reported a significant financial burden due to unreimbursed costs, with the mean monthly amount of unreimbursed expenses ranging from $262 to $682.

In terms of DEB, preliminary evidence exists from a study of 60 adult and children patients with recessive DEB (RDEB) in the UK [38]. The study revealed that there is a wide variation in costs related to dressing and retention garments for different subtypes of RDEB, with median costs ranging from £1699 (SD £2800) per year in RDEB inversa, to £85,156 (SD 68,875) per year in severe RDEB; costs for paid care were also the greatest for severe RDEB, which combined with dressing costs had a mean total of £97,943 per year. Another analysis from the same study revealed that there is a significant cost burden associated with wound care, with the average cost per year ranging from £2709 for RDEB inversa, to £81,858 for severe RDEB, with only 4 participants not requiring dressings [39]. Another recent small study in Ireland looking at children with moderate, mild, and very severe RDEB (n = 5, aged 2.5 to 10 years), revealed that total medical costs per year ranged from €7377 to €116,649, with the very severe group starting at €71,421, largely attributed to wound and drug costs [40].

### Study limitations

In terms of study limitations, attributing a HRQoL detriment to the underlying EB disease is complex due to the heterogeneity of severity across and within categories [1, 2, 6]. Dystrophic EB is a heterogenous disease, ranging from mild pretibial disease to life-threatening generalized recessive DEB, therefore the categorization of localized vs generalized might not be adequate to reflect the disease severity of this patient cohort. The Birmingham Epidermolysis Bullosa Severity (BEBS) score could have allowed for stratification of patients based on disease severity to provide more detailed cost estimates [11]. Although other EB (EBQoL) and dermatology-specific (Skindex-29) HRQoL patient reported outcome (PROM) instruments exist, the EuroQoL EQ-5D instrument was used as it is perceived to be a valid, generic PROM questionnaire that is widely applied in economic evaluations [7]. However, it should be noted that some disease features might not be adequately captured by such generic instruments, for which other disease-specific PROM tools could be explored [41].

Regarding the estimation of productivity losses, several methods exist [42]. Although the human capital approach used is grounded in economic theory, assuming companies employ labour until the marginal benefit of labour productivity equals the marginal cost of labour [43], in real world these assumptions might not hold; for example, a worker could be replaced on an interim basis by a less suitable person, or there might be a need for a new recruitment involving training costs [44]. Therefore, study results should be interpreted in alignment with the costing methods’ limitations.

Another limitation relates to sample selection. The relative small study sample and recruitment of patient volunteers could have introduced a selection bias in terms of patients’ severity, with implications for the comparison of results between the different countries; this is particularly relevant for Germany, which had a sample of 4 patients. For example, this could cause the inclusion of patients with relatively less severe illness, as they would be less likely to be hospitalised and available for study participation. In this scenario, the economic burden of the disease might have been underestimated, with the high costs for hospitalisation and long-term care not being captured.

On that front, another important limitation is that costs are likely to be underestimated because bandaging costs were not captured as part of medical costs. As suggested by other studies discussed above [38, 39], dressing, retention garments and wound care costs can be quite substantial, especially for severe patients. Therefore, if bandaging and related costs had been considered, it could be that the study’s conclusion about non-medical costs being the largest cost component, might have been different.

Furthermore, recall biases are non-negligible when conducting questionnaire-based studies and cross-sectional data was used; restrictions as to the scope and means of the study made the collection of longitudinal data prohibitive, although this could have captured patient adaptation to their diseased state [4].

Although some of these limitations might become encountered in cost-of-illness studies for prevalent diseases involving large patient samples, in smaller studies targeting rare disease populations such sample limitations are typically unavoidable and their implications should be carefully considered in the interpretation of results.

## Conclusion

By adopting a bottom-up, annual, socioeconomic approach, this study indicates the likely disease burden of DEB across five European countries. The study confirms earlier cost-of-illness evidence pointing towards a substantial negative impact on patient and caregiver HRQoL, in addition to economic implications which are predominantly attributable to high direct non-medical costs. Importantly, compared to the average EB patients, costs for DEB patients are higher across all main components of direct medical, direct non-medical, and indirect costs.

## Supplementary Information

**Additional file 1.** Generic questionnaire instrument used for data collection.

**Additional file 2.** Medical unit cost sources.

**Additional file 3. Table S1:** Average annual costs per patient (SD), localised patients (n = 21, 2020 €) and **Table S2**: Average annual costs per patient (SD), generalised patients (n = 70, 2020 €).

## Data Availability

The data that support the findings of this study are available from the authors upon reasonable request and with permission of Krystal Biotech.
