# Supplementary material for: Understanding the socioeconomic costs of dystrophic epidermolysis bullosa in Europe: a costing and health-related quality of life study
Source: Orphanet J Rare Dis. 2022 Sep 6;17:346. doi: 10.1186/s13023-022-02419-1 (PMC9450448; doi:10.1186/s13023-022-02419-1)
Supplement: Supplementary file 1 — Additional file 1. Generic questionnaire instrument used for data collection. [file 13023_2022_2419_MOESM1_ESM.pdf]

Annexes

Annex I: Initial translation of the questionnaire to English (general version for all diseases)

A) Patient's questionnaire

NAME OF THE DISEASE

BURQOL RD

Questionnaire for the patient over 17

Sub-type of the disease – only where applicable

____

1. **Patient's age** _____
2. **Sex** o Female o Male
3. **Region** _______________
4. **Marital status**
   o Single
   o Separated
   o Married or cohabiting
   o Widow/er
   o Divorced
5. **Level of studies completed**
   o Primary o o University
   o Secondary o o None
6. **Number of household members where the patient lives:** ___ people
7. **At what age were you diagnosed with the disease?** ___ years
8. **Carer**
   a. **Do you need a carer to assist you with your daily activities?** (for basic hygiene, to help you to move, administration of drugs, performing treatments, etc.)
      o Yes o No ⇒ Skip to question 9
   b. **In the case of yes, who is your <u>principal carer</u>?**
      o Family member ⇒ Fill in the carer questionnaire
      o Another non-contracted person (friend) ⇒ Fill in the carer questionnaire
      o Professional carer (contracted or provided by an entity)
   c. **If you use the services of a <u>professional carer</u>,**
      How many hours a week? ______ hours a week
      Who pays for the service and how much?
      o I pay the entire cost of ______ € per hour
      o The cost is covered by social security or another entity
      o The cost is partially covered by social security or another entity, I pay____ € per hour
9. **What is your working situation?**
   o I am employed
   o Student ⇒ Skip to question 12
   o I am unemployed
   o Temporarily on leave of absence
   o Permanent work disability ⇒ Skip to question 11
   o Retired ⇒ Skip to question 11

DRAFT

1

o Domestic tasks ⇒ Skip to question 12

o Special situation (occupational workshops)

10. Work limitation (only to be answered if the patient is working or on temporary leave of absence)

a. Has the fact you suffer from the disease meant any work-related problem in the last 6 months?

o Yes o No ⇒ Skip to question 12

b. In case of yes, please specify this

o I was off work ___ days

o I was working ___ hours less a day for ___ days

o I am working ___ hours less a day

o I do not work less hours a day but I have problems performing

o Other problems: ________________________________

⇒ Skip to question 12

11. Abandonment of work (only to be answered in case the patient is retired or in a situation of permanent work disability)

a. Have you had to abandon your job or retire early because of your disease?

o Yes o No ⇒ Skip to question 12

o I have not been able to work as a consequence of my disease ⇒ Skip to question 12

b. In case of yes, please specify in what way

o I had to abandon my job at the age of ___ years

o I had to retire early at the age of ___ years

12. Do you have the disability certificate and its degree?

o Yes ⇒ Please indicate the degree: ___

o I have requested this and it is being processed

o I have requested this but it was refused (under 33%)

o I do not have one and I have not requested this

o Not applicable (the affected party is not old enough)

13. Do you have the dependence assessment?

o Yes, I have the assessment now ⇒ Please specify: Degree ___ Level ___

o I have requested this, it is being processed

o No, but I intend to request this

o I have no intention of requesting this

14. Which drugs have you taken in the last month (because of your disease)? Please select the drugs and specify the form of payment.

If one of the drugs is not on the list, please specify this to us by writing its name.

Commercial drug name (active substance)	Cost covered by the health system

DRAFT

NAME OF THE DISEASE

BURQOL RD

		Yes	No	Partial
❑		❑	❑	❑
❑		❑	❑	❑
❑		❑	❑	❑
❑		❑	❑	❑

15. To which medical tests or examinations prescribed by a doctor, have you been subjected in the last 6 months (because of your disease)? *If any of the tests are not on the list, please specify this to us.*

	Medical tests or examinations	No of times in 6 months	Cost covered by the health system		
			Yes	No	Partial
❑			❑	❑	❑
❑			❑	❑	❑
❑			❑	❑	❑
❑			❑	❑	❑

16. How many visits to specialists have you had to undergo in the last 6 months? *If any of the specialists are not on the list, please specify this to us.*

	Specialist	No of times in 6 months	Cost covered by the health system		
			Yes	No	Partial
❑	Genetic counselling		❑	❑	❑
❑	Cardiologist		❑	❑	❑
❑	Surgeon		❑	❑	❑
❑	Dermatologist		❑	❑	❑
❑	Gastroenterologist		❑	❑	❑
❑	Endocrinologist		❑	❑	❑
❑	Physiotherapist		❑	❑	❑
❑	Gynaecologist		❑	❑	❑
❑	Haematologist		❑	❑	❑
❑	Immunologist		❑	❑	❑
❑	Speech therapist		❑	❑	❑
❑	Nephrologist		❑	❑	❑
❑	Pulmonologist		❑	❑	❑
❑	Neurosurgeon		❑	❑	❑
❑	Neurologist		❑	❑	❑
❑	Dentist		❑	❑	❑
❑	Ophthalmologist		❑	❑	❑
❑	Oncologist		❑	❑	❑
❑	Otolaryngologist		❑	❑	❑
❑	Chiropodist		❑	❑	❑
❑	Psychologist		❑	❑	❑

DRAFT

❑	Psychiatrist		❑	❑	❑
❑	Rheumatologist		❑	❑	❑
❑	Traumatologist		❑	❑	❑
❑	Urologist		❑	❑	❑
❑			❑	❑	❑
❑			❑	❑	❑

17. How many visits to the <u>GP</u> / <u>Nurse</u> / <u>Casualty</u> / have you had to undergo in the last <u>6 months</u>?

o GP	___ visits to the health centre<br>___ domiciliary visits
o Nurse	___ visits to the health centre<br>___ domiciliary visits
o Casualty	___ visits to the health centre<br>___ domiciliary visits<br>___ visits to the hospital

18. Hospitalisation

a. How often and how many days have you had to <u>be admitted to hospital</u> in the last <u>12 months</u> (because of your disease)?

___ times ___ days in total

b. During the hospital admissions in the last 12 months, have you undergone <u>lung transplant</u>? (only for Cystic Fibrosis)

o Yes o No

19. Please specify the <u>health material</u> you have had to use in the last <u>6 months</u>. *If any of the health materials are not on the list, please specify this to us.*

	Health material	Cost covered by the health system		
		Yes	No	Partial
❑		❑	❑	❑
❑		❑	❑	❑
❑		❑	❑	❑
❑		❑	❑	❑

20. What difficulties have you had to obtain health products?

o Lack of product stock

o The product has been withdrawn

o It has to be obtained in another country

o It is indicated for another pathology but not for mine

o Product too expensive

o Unsuitable pharmaceutical formula

o Other difficulties (please specify): ______________________________

21. How many times have you used *means of transport* in the last 6 months for disease-related travel to the health centre, hospital, rehabilitation, etc.?

	Kind of transport	No of times in 6 months	Cost covered by the health system		
			Yes	No	Partial
❑	Private car		❑	❑	❑
❑	Taxi		❑	❑	❑
❑	Bus/Train		❑	❑	❑
❑	Aeroplane		❑	❑	❑
❑	Health and/or adapted transport		❑	❑	❑
❑	Ambulance		❑	❑	❑

22. Health and social services you have required and received, their financial system and the reasons for which you have not received the services you require.

Mark the services you have required, number of days received and form of payment. In case you have required a service and have not received it, note the reason number (see the possible reasons below the table).

Kinds of services	Because of their disease they have needed to receive...	Days service received according to economic system.			Reason for which they have not received the service (*)
		Free of charge	Direct payment	Mixed (public and private)	
During the last month...		During the last month...			
1. Telecare	❑	__ days	__ days	__ days	
2. Programmed domiciliary care	❑	__ days	__ days	__ days	
3. Social domiciliary help	❑	__ days	__ days	__ days	
4. Day centre	❑	__ days	__ days	__ days	
5. Occupational centres	❑	__ days	__ days	__ days	
6. Cultural, recreational and leisure and free time activities	❑	__ days	__ days	__ days	
7. Other:...	❑	__ days	__ days	__ days	
In the last 6 months...		In the last 6 months...			
8. Occupational therapy and/or training in ADLs (Activities of Daily Living)	❑	__ days	__ days	__ days	
9. Information/Advice/Assessment	❑	__ days	__ days	__ days	
10. Psychosocial care to family members	❑	__ days	__ days	__ days	
11. Respite services: Temporary stays	❑	__ days	__ days	__ days	
12. Services from sign language interpreters	❑	__ days	__ days	__ days	
13. Other alternative communication systems	❑	__ days	__ days	__ days	
14. Residential centres	❑	__ days	__ days	__ days	
15. Tourism and hydrotherapy for disabled people	❑	__ days	__ days	__ days	
16. Work orientation/preparation	❑	__ days	__ days	__ days	
17. Other:...	❑	__ days	__ days	__ days	

(*) Reasons: 1 – Waiting list. 2 – Not available in the setting. 3 – Cannot pay it. 4 – Does not comply with any of the requirements. 5 – Other reasons.

23. Are you satisfied with the health care received because of your disease?
Please indicate the degree of your satisfaction on a scale of 1 to 10.

☐	☐	☐	☐	☐	☐	☐	☐	☐	☐
**1**	**2**	**3**	**4**	**5**	**6**	**7**	**8**	**9**	**10**

Not at all satisfied.. Very satisfied

24. Who answers the questionnaire for the patient?

o The person with a rare disease on their own

o The person with a rare disease with the support of an informant or interpreter

o An informant (family member, tutor, carer, etc.)

EQ-5D-5L (5 levels)

Barthel Index

B) Carer's questionnaire

BURQOL RD

QUESTIONNAIRE FOR THE CARER

The patient's principal informal carer (not contracted) fills in the questionnaire

1. **Carer's age** ____
2. **Sex** o Female o Male
3. **Region** ______________
4. **Marital status**
   - o Single
   - o Separated
   - o Married or cohabiting
   - o Widow
   - o Divorced
5. **What is your relationship with the patient? Are you their.**
   - o Mother/father
   - o Grandparent
   - o Uncle
   - o Another (please specify) ________
   - o Sibling
6. **Since when have you cared for the patient?** ______years ______months
7. **What is your working situation?**
   - o I am employed
   - o Retired or pensioner ⇒ Skip to question 9
   - o Domestic tasks ⇒ Skip to question 10
   - o Other (student, unemployed, etc.) ⇒ Skip to question 10
8. **Work-related problem (only to be answered in case the carer is active (employed or unemployed)**
   - a. **Has caring for the patient because of the disease they suffer meant any work-related problem for you in the last 12 months?**
     o Yes o No ⇒ Skip to question 10
   - b. **In case of yes, please specify this**
     - o I requested___ days leave of absence
     - o I was working ___ hours less a day for ___ days
     - o I am working ___ hours less a day
     - o I do not work less hours a day but I have problems fulfilling my working hours
     - o Other problems: ____

⇒ Skip to question 10

9. **Retirement (only to be answered in case the carer is retired or a pensioner)**
   - a. **Have you had to retire early to care for the patient?**
     o Yes o No ⇒ Skip to question 10
   - b. **In case of yes, please specify:**
     o I retired early at the age of___ years

10. ZARIT Scale

11. EQ-5D-5L (5-level)

DRAFT 13

QUESTIONNAIRE FOR THE CARER

Regarding the role played by you as PRINCIPAL CARER...

12a. How much time do you invest in a normal DAY for each one of the activities related to the patient's disease?
Please specify the approximate time you spend daily on each activity

On basic hygiene and dressing or changing them	______	hours	______	minutes	a day
On bathing or showering them	______	hours	______	minutes	a day
On feeding them	______	hours	______	minutes	a day
On helping them to move	______	hours	______	minutes	a day
On cooking and preparing meals	______	hours	______	minutes	a day
Administration of drugs	______	hours	______	minutes	a day

12b. How much time do you invest in a normal WEEK on each one of the following activities related to the patient's disease?
Please specify the approximate time you spend weekly on each activity

On domestic tasks (cleaning, laundry, etc...)	______	hours	______	minutes	a week
On travel	______	hours	______	minutes	a week
On shopping	______	hours	______	minutes	a week
On financial, administrative or legal affairs	______	hours	______	minutes	a week
On social and leisure activities	______	hours	______	minutes	a week
Monitoring and supervision (falls)	______	hours	______	minutes	a week

Regarding the role played by OTHER CARERS (e.g. the rest of the family)

13a. How much time in a normal DAY do other carers spend on each one of the following activities related to the patient's disease?
Please specify the approximate time these people spend daily on each activity

On basic hygiene and dressing or changing them	______	hours	______	minutes	a day
On bathing or showering them	______	hours	______	minutes	a day
On feeding them	______	hours	______	minutes	a day
On helping them to move	______	hours	______	minutes	a day
On cooking and preparing meals	______	hours	______	minutes	a day
Administration of drugs	______	hours	______	minutes	a day

13b. How much time do other carers spend in a normal WEEK on each of the following activities related to the patient's disease?
Please specify the approximate time these people spend weekly on each activity

On domestic tasks (cleaning, laundry, etc...)	______	hours	______	minutes	a week
On travel	______	hours	______	minutes	a week
On shopping	______	hours	______	minutes	a week
On financial, administrative or legal affairs	______	hours	______	minutes	a week
On social and leisure activities	______	hours	______	minutes	a week
Monitoring and supervision (falls)	______	hours	______	minutes	a week

DRAFT

14
