# Supplementary material for: Understanding the socioeconomic costs of dystrophic epidermolysis bullosa in Europe: a costing and health-related quality of life study
Source: Orphanet J Rare Dis. 2022 Sep 6;17:346. doi: 10.1186/s13023-022-02419-1 (PMC9450448; doi:10.1186/s13023-022-02419-1)
Supplement: Supplementary file 3 — Additional file 3. Table S1: Average annual costs per patient (SD), localised patients (n = 21, 2020 €) and Table S2: Average annual costs per patient (SD), generalised patients (n = 70, 2020 €). [file 13023_2022_2419_MOESM3_ESM.docx]

**Table S1: Average annual costs per patient (SD), localised patients (n = 21, 2020 €)**

| Localised | **Average** | | **France** | | **Germany** | | **Italy** | | **Spain** | | **United Kingdom** | |
| --- | --- | --- | --- | --- | --- | --- | --- | --- | --- | --- | --- | --- |
|  | Mean | SD | Mean | SD | Mean | SD | Mean | SD | Mean | SD | Mean | SD |
| Drugs | 433 | 1,077 | 63 | 154 | N/A | N/A | 2,881 | 1,700 | 178 | 165 | 52 | 64 |
| Tests | 139 | 178 | 162 | 130 | N/A | N/A | 126 | 219 | 220 | 222 | 85 | 209 |
| Visits | 2,081 | 4,107 | 1,668 | 850 | N/A | N/A | 1,512 | 2,005 | 677 | 861 | 5,126 | 7,647 |
| Hospitals | 3,284 | 10,541 | 479 | 525 | N/A | N/A | 12,482 | 21,620 | 6,416 | 15,249 | 0 | 0 |
| Material | 1,245 | 1,374 | 1,574 | 1,857 | N/A | N/A | 382 | 630 | 24 | 23 | 2,428 | 527 |
| HC Transport | 7 | 36 | 29 | 72 | N/A | N/A | 0 | 0 | 0 | 0 | 0 | 0 |
| **Direct Medical** | 7,190 | 12,156 | 3,976 | 2,717 | N/A | N/A | 17,384 | 25,181 | 7,514 | 16,409 | 7,691 | 7,485 |
| Social health service | 310 | 1,100 | 893 | 2,103 | N/A | N/A | 110 | 191 | 0 | 0 | 0 | 0 |
| Professional Carer | 136 | 666 | 544 | 1,332 | N/A | N/A | 0 | 0 | 0 | 0 | 0 | 0 |
| Non-HC Transport | 122 | 217 | 118 | 176 | N/A | N/A | 300 | 505 | 75 | 132 | 64 | 124 |
| Main informal carer | 19,091 | 29,589 | 1,197 | 2,931 | N/A | N/A | 13,916 | 24,103 | 23,060 | 26,723 | 40,760 | 44,651 |
| Other informal carer | 2,799 | 6,871 | 0 | 0 | N/A | N/A | 3,340 | 5,786 | 6,049 | 11,739 | 2,548 | 6,240 |
| Informal care total | 21,891 | 33,594 | 1,197 | 2,931 | N/A | N/A | 17,256 | 29,889 | 29,109 | 35,953 | 43,308 | 47,769 |
| **Direct Non-medical** | 22,459 | 33,458 | 2,751 | 4,906 | N/A | N/A | 17,666 | 30,584 | 29,185 | 35,910 | 43,372 | 47,849 |
| Productivity loss | 772 | 3,338 | 2,716 | 6,654 | N/A | N/A | 746 | 1,293 | 0 | 0 | 0 | 0 |
| Early retirement | 4,292 | 11,028 | 0 | 0 | N/A | N/A | 2,712 | 4,698 | 3,483 | 8,531 | 12,330 | 19,102 |
| **Indirect** | 5,065 | 11,218 | 2,716 | 6,654 | N/A | N/A | 3,459 | 4,204 | 3,483 | 8,531 | 12,330 | 19,102 |
| **TOTAL** | 34,713 | 44,256 | 9,443 | 8,601 | N/A | N/A | 38,510 | 59,821 | 40,181 | 47,257 | 63,394 | 56,073 |

**Table S2: Average annual costs per patient (SD), generalised patients (n = 70, 2020 €)**

| Generalised | **Average** | | **France** | | **Germany** | | **Italy** | | **Spain** | | **United Kingdom** | |
| --- | --- | --- | --- | --- | --- | --- | --- | --- | --- | --- | --- | --- |
|  | Mean | SD | Mean | SD | Mean | SD | Mean | SD | Mean | SD | Mean | SD |
| Drugs | 1,205 | 1,780 | 26 | 48 | 60 | 77 | 3,419 | 1,628 | 397 | 687 | 76 | 85 |
| Tests | 220 | 441 | 95 | 89 | 131 | 218 | 67 | 98 | 288 | 457 | 686 | 845 |
| Visits | 2,562 | 3,972 | 1,089 | 2,427 | 5,240 | 4,084 | 2,389 | 2,132 | 3,100 | 5,741 | 2,957 | 2,855 |
| Hospitals | 2,867 | 6,103 | 3,236 | 6,739 | 6,026 | 9,564 | 3,062 | 5,931 | 2,869 | 6,591 | 2,210 | 5,221 |
| Material | 1,015 | 1,628 | 2,473 | 1,871 | 617 | 1,101 | 645 | 1,379 | 516 | 1,547 | 2,552 | 1,242 |
| HC Transport | 86 | 427 | 0 | 0 | 502 | 581 | 0 | 0 | 151 | 668 | 58 | 175 |
| **Direct Medical** | 7,955 | 9,258 | 6,919 | 10,240 | 12,576 | 14,221 | 9,582 | 7,674 | 7,321 | 10,640 | 8,540 | 7,390 |
| Social health service | 1,909 | 6,223 | 4,678 | 12,634 | 7,699 | 15,398 | 2,128 | 5,361 | 814 | 2,195 | 0 | 0 |
| Professional Carer | 661 | 4,176 | 0 | 0 | 0 | 0 | 642 | 2,127 | 0 | 0 | 3,871 | 11,614 |
| Non-HC Transport | 123 | 282 | 269 | 655 | 466 | 604 | 63 | 74 | 100 | 150 | 52 | 81 |
| Main informal carer | 30,053 | 34,819 | 8,834 | 18,190 | 40,237 | 46,502 | 30,767 | 35,847 | 46,071 | 35,209 | 8,236 | 21,311 |
| Other informal carer | 11,517 | 20,338 | 5,087 | 10,937 | 8,598 | 14,984 | 14,938 | 22,708 | 15,592 | 24,386 | 2,952 | 8,856 |
| Informal care total | 41,570 | 49,122 | 13,921 | 29,114 | 48,835 | 57,150 | 45,705 | 51,100 | 61,662 | 51,597 | 11,188 | 30,095 |
| **Direct Non-medical** | 44,263 | 49,554 | 18,869 | 42,171 | 57,000 | 69,348 | 48,538 | 49,424 | 62,577 | 50,727 | 15,111 | 30,719 |
| Productivity loss | 86 | 637 | 0 | 0 | 0 | 0 | 0 | 0 | 209 | 1,066 | 110 | 330 |
| Early retirement | 2,721 | 8,801 | 0 | 0 | 9,829 | 19,658 | 708 | 2,344 | 2,864 | 8,145 | 8,220 | 16,312 |
| **Indirect** | 2,806 | 8,797 | 0 | 0 | 9,829 | 19,658 | 708 | 2,344 | 3,074 | 8,139 | 8,330 | 16,253 |
| **TOTAL** | 55,024 | 53,422 | 25,787 | 51,846 | 79,405 | 72,302 | 58,827 | 49,389 | 72,971 | 55,327 | 31,981 | 41,097 |
